# Comparison of Emergency Department Boarders Managed by a Critical Care Consult Service versus Standard Care

**DOI:** 10.5811/westjem.53002

**Published:** 2026-06-28

**Authors:** Matthew Johnson, Alexander Bracey, Ashar Ata, William DaSilva, Sean Patrick Geary, Iman Aly, Warren Hayashi, Rachel Leavitt, James Mantas, Christopher Hanowitz, Luke Duncan, Denis Pauze, Gregory P. Wu

**Affiliations:** Albany Medical Center, Department of Emergency Medicine, Albany, New York

## Abstract

**Introduction:**

Emergency department (ED) boarding of critically ill patients awaiting intensive care unit (ICU) admission has been associated with delays in time-sensitive interventions, prolonged lengths of stay (LOS), increased crowding, and higher incidence of morbidity and mortality. To address this, our institution implemented an ED critical care consult team in 2020 and established a resuscitation and emergency critical care fellowship in 2022. The critical care consult team, staffed by emergency physicians with additional resuscitation or critical care training, provides consultative support for resuscitations and the management of boarding critically ill patients 10 hours per day, with the dual goals of improving clinical care and optimizing patient flow. The primary outcome of this study was to evaluate the rate of patients downgraded from their initial admission level of care (ICU, Intermediate care) to a lesser intensity of care (intermediate, ward, home). Secondary outcomes included ED, ICU, and hospital LOS.

**Methods:**

We conducted a retrospective chart review of all patients ≥ 18 years old with ICU or intermediate care admission orders placed between November 1–30, 2024. Patients were sequentially identified via the electronic health record, and clinical and quality assurance data were collected in a REDCap database.

**Results:**

During the study period, the ED critical care consult team was active on 73% of days, staffed primarily by resuscitation and emergency critical care fellows. Of 372 eligible patients, 18% (68/372) were managed by the critical care consult team after initial assessment by primary emergency physician staff, while 82% (304/372) served as controls and did not have involvement with the critical care consult team. Patients evaluated by the team had higher acuity, with 30.9% presenting with an Emergency Severity Index (ESI) score 1 compared to 4.6% in the control group. Despite this, the downgrade rate was significantly higher in the ED3CT cohort (29.4% vs 11.8%, p=0.001). No differences were observed in ED (0.62 days vs 0.60 days, *P* = .80), ICU (5.1 days vs. 5.2 days, *P* = .93), or hospital LOS (9.7 days vs 10.8 days, *P* = .68).

**Conclusion:**

These findings suggest that ED-based critical care physicians, including fellows in training, may facilitate earlier identification of patients suitable for lower levels of care despite initially higher acuity. While there was no change in ED, ICU, and hospital length of stay, it is possible that this is related to systemic problems with hospital bed availability. The high rate of downgrades highlights the benefits of ED-based critical care physicians advancing care of critically ill patients boarding in the ED.

## INTRODUCTION

The practice of emergency department (ED) boarding is among the most pressing issues in emergency medicine, notably in regard to critically ill patients waiting for an intensive care unit (ICU) bed. Prior work has demonstrated an increased number of critically ill ED patients in the last decade without a concomitant increase in the number of ICU beds.^1^,^2^ Critically ill patients boarding in the ED while awaiting ICU admission experience prolonged hospital length of stay (LOS), delays in time-sensitive interventions, increased crowding-related system strain, and higher risks of morbidity and mortality due to suboptimal nurse-to-patient ratios, reduced multidisciplinary ICU involvement, and inconsistent implementation of standardized critical care pathways.^3^ Various approaches have been employed to address these issues, including early identification systems, protocolized treatment plans, ED-based critical care teams or units, telemedicine support, and hastening ICU admissions.^3^–^5^ However, these approaches tend to be specific to the institution in which they are developed and lack generalizability and consistency of benefit.

We have previously described our local approach to this challenge and its impact on financial outcomes; in this study we describe the effects on clinical outcomes.^6^ At our institution (an academic, urban, quaternary referral center), we instituted the ED critical care consult team in 2020 and established a resuscitation and emergency critical care fellowship in 2022.^7^ Our adult ED has approximately 60,000 patient visits per year and is licensed for 73 acute care beds. Intensive care and intermediate care (also known as high-dependency care, stepdown care, or progressive care) admissions make up to 16% of annual visits. The ED critical care consult team is composed of emergency physicians with additional critical care training (critical care medicine, surgical critical care or resuscitation, and emergency critical care fellowships). This team assists with the management of critically ill patients boarding within the ED and active resuscitations.

The goal of our ED critical care consult team is multifactorial. Its focused delivery of critical care to patients boarding in the ED while awaiting placement in an ICU or intermediate care unit bed frees the broader ED to focus on other tasks. Implementation of similar programs, such as the Stanford Emergency Critical Care Program, has led to decreases in in-hospital mortality.^8^ In addition to improved clinical outcomes, one of the primary goals of the team is to improve flow through the ED. The primary outcome of this study was to evaluate the downgrade rate of patients managed by the ED critical care consult team—meaning those patients who received a downgrade order to a lesser intensity of care (ie, ICU-to-intermediate care or intermediate care-to-ward). We ultimately chose the downgrade rate because it is a marker of clinical improvement in a patient’s status, as well as an indication that the patient is less of a burden on the overall ED staff. Secondary outcomes included ED LOS, hospital LOS, and ICU LOS.

**Population Health Research Capsule**

What do we already know about this issue?

*Intensive care unit boarding in the ED is linked to delays in care, longer length of stay (LOS), and increased mortality among critically ill patients*.

What was the research question?

*Does an ED critical care team increase ICU downgrades vs standard care?*

What was the major finding of the study?

*Downgrades were 3-fold higher with an ED critical care team (29.4% vs 11.8%, P = .001); ED LOS was unchanged*.

How does this improve population health?

*An ED critical care team improves ICU resource use and patient flow, reducing boarding strain and supporting safer ED care delivery*.

## METHODS

We conducted a retrospective chart review of patients for whom admission orders to an ICU or intermediate care unit were placed (ie, intended for admission) between November 1–November 30, 2024. All patients were ≥ 18 years of age. The study was approved by the institutional review board (STUDY00004598), and records were reviewed in accordance with best practices for emergency medicine research.^9^ Patients were sequentially identified by a single author through a query of the electronic health record (Epic Systems Corporation, Verona, WI). We collected data using a predefined abstraction to capture demographic, primary, and secondary outcome measures. Extracted data were entered into a secure REDCap database (Research Electronic Data Capture hosted at Albany Medical Center). We used medical record numbers and individual encounter numbers to link admission orders with corresponding demographic and outcome data. The abstractor was not blinded to the study hypothesis, and performance was not monitored. Because there was a single abstractor, interrater reliability was not assessed, and duplicate sampling was not performed. There were no missing data.

Within our institution, the decision to admit to a specific level of care is made via conversation between the emergency physician and the accepting service, with specific indications noted in Table 1. The ICU subtypes included medical, surgical, neuroscience, cardiac, and cardiovascular surgical. The intermediate care subtypes included medical, surgical, neuro, cardiac, and spine intermediate care units. The ED critical care care team was not involved in the initial decision to admit to a specific level of care.

We summarized categorical variables as frequency counts and percentages and compared them via Pearson chi-square and Fisher exact test, as appropriate. Continuous variables were summarized as means and standard deviations and were compared via *t*-tests and Wilcoxon rank-sum tests, as appropriate. We used statistical software STATA 18.0 (StataCorp, LLC, College Station, TX) for analysis.

## RESULTS

**Figure 1:**
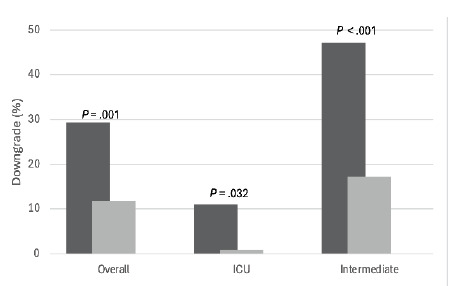
Downgrade rates separated into initial level of care for patients managed by the emergency department critical care consult team and the control group in a prospective review of critically ill patients boarding in the emergency department. *ED3CT*, emergency department critical care consult team; *ICU*, intensive care unit*; IMC*, intermediate care unit.

During the study period, the ED critical care consult team was active on 22 of 30 days (73%) between 2 pm - 12 am. All shifts were primarily staffed by resuscitation and emergency critical care fellows with backup on-call coverage available from the emergency physician critical care leadership. We identified 372 patients intended for ICU or intermediate care unit admission. Of those patients, 68 of 372 (18%) were managed by the ED critical care consult team, while 304 of 372 (82%) had no contact with the team and served as the control group. When comparing patients between the critical care consult team and control groups, we noted similar ages and primary ED diagnoses (Table 2). However, the rates of respiratory distress and sepsis were higher in the ED critical care consult team group compared with the control group. In contrast, the control group had high rates of neurologic and cardiac emergencies. In general, the patients seen by the ED critical care physician had higher acuity, with > 30% of patients having an initial ESI score of one compared to < 5% in the control group. This was further demonstrated by the initial disposition level of intensity, with trends toward higher initial dispositions of ICU level of care being seen by the ED critical care consult physician (39.7% and 31.9%, respectively). Despite the overall higher acuity, the downgrade rates with the team were 29.4% versus 11.8% with the control group. This finding persisted when controlling for the initial disposition intensity of care (Figure 1). However, despite these higher rates of downgrades, we found no differences in ED, hospital, or ICU LOS.

## DISCUSSION

Our findings add to the growing evidence in support of the utility of ED-based critical care consult physicians. We found that patients seen by these emergency physicians were three times more likely to be downgraded to a lesser intensity of care. This finding persisted across both ICU and intermediate care unit levels of care, despite minor differences in patient populations that may reflect the ED critical care consult team’s predilection for managing the sickest patients in the ED. This selection bias may have contributed to the observed similarity in hospital courses (both ICU/intermediate care unit and overall) between cohorts, despite a significant increase in patient downgrades with the critical care team cohort. These findings are similar to those reported by Gunnerson et al after the introduction of an emergency critical care center and Mitarai et al after the implementation of an emergency critical care program. Both observed a reduction in ICU admissions following their approaches to ED-based critical care.^8^,^10^ However, both studies used before-and-after comparisons, while our data are part of a sequentially enrolled cohort study.

The effects of boarding on critically ill patients have been well defined in the literature since the work of Chalfin et al showed an increase in mortality after six hours of boarding.^11^ In addition to the effects on the individual patient, prolonged ED boarding has effects across the healthcare system, including prolonged ED LOS for all patients, higher numbers of left before completion of care, and increased stressors on individual clinicians.^3^ The introduction of this team has nearly tripled the downgrade rate among critically ill patients boarding in the ED. Although it is a surrogate outcome, transitioning patients from the ICU or intermediate care unit to a lower level of care may serve as a marker of clinical improvement as well as reduced nursing and departmental intensity of care (ie, less frequent nursing checks, medication titrations, ventilatory support monitoring, etc).

Our data are likely the first to explore the impact of a resuscitation and emergency critical care fellowship on departmental throughput and ED-based critical care, as all ED critical care consult team shifts during this period were covered by resuscitation and emergency critical care fellows with backup from emergency physician critical care attending. To our knowledge, prior work has been exclusively focused on the attending-level care. While this limits the generalizability of our data, it further reinforces the potential benefits of emergency physicians with additional critical care training and focus on the ED. It is plausible that our results underestimate the overall benefits of the ED critical care consult physician, given the difference in acuity between our groups, as well as the clinicians providing the direct bedside care still being in training.

Finally, despite the significant downgrade rate, we did not see any significant changes in ED LOS or ICU LOS. This likely owes to our institution typically operating at or above capacity. Importantly, downgrading patients who were boarding in the ED for an ICU or intermediate care unit bed did not lengthen the ED course, but did offload the ICU, potentially creating additional capacity for those who needed this scarce resource.

## LIMITATIONS

There are several limitations to this report. First, these data are from a single center over one month. Second, our division has five emergency physicians with additional formal critical care fellowship training and a resuscitation and emergency critical care fellowship, which may limit the generalizability of these findings. Third, there are differences between our two patient populations, which are likely multifactorial and related to the methods of ED critical care team consultation, day-to-day variations in ICU boarding, and department-specific needs. This potentially introduces selection bias, although we reviewed all patients during the study period and found our results held across multiple subgroups. Fourth, we used the ESI to compare acuity and resource use between the control and intervention groups. However, the ESI is ultimately a triage tool that was developed to identify acuity on arrival to the ED and resource use, and not the severity of critical illness as do the APACHE or sequential organ failure assessment scores.

We do think that when considering patient ED boarding and resource use, the ESI has a role, but we acknowledge that it is not the best marker of overall patient acuity. Fifth, the specific indications for ICU and intermediate care unit admission vary between institutions (our specific indications are noted in Table 1). Ours requires admission for respiratory failure on no-invasive ventilatory support, as well as diabetic ketoacidosis requiring continuous insulin infusion and hourly finger sticks that may not require higher levels of care at other institutions. Ultimately, this leads to some system-specific benefits, and it is unclear what portion of our downgrades came from these populations (general diagnosis seen in Table 2). Nevertheless, we did not see changes in ED LOS; the prevention of ICU/intermediate care unit admissions for patients with frequent short stays in the ICU has previously been associated with improved ICU bed use for more complex patients.^12^

Sixth, these patients were seen by resuscitation and emergency critical care fellows primarily with attending-level oversight; we had no control group of patients re-evaluated by an emergency physician separate from evaluation by the ED critical care team. This raises the question about what specific level of clinician (ie, resident, fellow, general emergency physician, ED critical care physician, etc) is required to show benefit. However, patients are managed continuously throughout their ED stay by the general emergency physicians, and the downgrade rate is substantially lower than that seen by the ED critical care consult team. Whether this is due to comfort with transition in care by this team (resuscitation and emergency critical care fellows and ED critical care physicians) is not identified in this study.

Seventh, we did not record the length of time needed for each ED critical care team consultation; we estimate 30–60 minutes per patient, but this is purely anecdotal. Ultimately, we are reassured that despite this additional consultation, the ED LOS did not change. Eighth, at our institution, it is the responsibility of the emergency physician, rather than the intensivist, to assess for downgrades; this makes downgrades an emergency physician-driven process as opposed to an intensivist-driven process. This may limit generalizability at institutions where intensivist-driven downgrades are common. However, the significant benefit of a dedicated ED-based critical care physician is reflected in our data, as patients seen by the ED critical care consultation team were three times more likely to be downgraded than those who were not evaluated by the team. Further work is needed to evaluate whether this benefit is specific to ED-based critical care physicians, ICU physicians, or other clinicians.

## CONCLUSION

Patients seen by an ED critical care consult physician had a three times higher downgrade rate when compared to the control group. Given the significant stressors that critically ill patients boarding in the ED place on the department as a whole, we feel that targeted care to these patients with ED-based critical care physicians may have a growing role to play in the care of the sickest patients in the ED in the coming years.

## Figures and Tables

**Table 1:** List of criteria for admission to intensive care and intermediate care units at our institution.

	ICU Admission	IMC admission
Nursing or Lab Intervention	Every hour intervention	Every 2–4 hour intervention
Vasopressors	Greater than 10 μg/min of norepinephrine or equivalent	< 10 μg/min of norepinephrine or equivalent
Inotropes/inodilators	yes	no
Multiple vasoactive medications	yes	no
Continuous analgosedation	yes	yes
Continuous electroencephalogram	yes	yes
Respiratory support	Invasive ventilation	High-flow oxygen and/or noninvasive positive pressure ventilation
Extracorporeal therapy	yes	no
	Clinician Discretion	

Vasopressors include norepinephrine, phenylephrine, and vasopressin. Inotropes and inodilators include epinephrine, dobutamine, dopamine, and milrinone. Continuous analgosedation permitted in the IMC is limited exclusively to dexmedetomidine. Extracorporeal therapies include CRRT, microaxial flow pump, IABP, and ECMO. All admission decisions are ultimately at the clinician’s discretion.

*CRRT*, continuous renal replacement therapy; *ECMO*, extracorporeal membrane oxygenation; *ICU*, intensive care unit; *IMC*, intermediate care unit; *IABP*, intra-aortic balloon pump; *μg*, microgram.

**Table 2:** Demographic data and outcomes comparing patients managed by the emergency department critical care team and the control group in a prospective review of critically ill patients boarding in the emergency department.

	ED3CT (68)	Control (304)	*P* value
Age (years)	60	65	.04
ESI (%)			
1	30.9	4.6	
2	58.8	72.0	
3	8.8	19.7	
unknown	1.5	3.6	< .001
Primary ED diagnosis (%)			
Trauma	22.1	20.7	
Stroke	14.7	21.4	
Respiratory disease	13.2	7.9	
Sepsis	13.2	6.6	
Cardiac	2.9	8.6	
Other	33.8	34.9	.66
Initial Level of Care			
Ward	7.3	1.6	
IMC	52.9	66.4	
ICU	39.7	31.9	.09
Emergency department length of stay (days)	0.62	0.60	.80
Hospital length of stay (days)	9.7	10.8	.68
ICU length of stay (days)	5.1	5.2	.93

*ED3CT*, emergency department critical care consult team: *ESI*, Emergency Severity Index; *IMC*, intermediate care unit, *ICU*, intensive care unit.
